# Assessment of primary patency for femoropopliteal graft entrapment within the sartorius muscle

**DOI:** 10.1007/s00423-024-03470-1

**Published:** 2024-09-17

**Authors:** Jeffrey Rodgers, Thomas E. Brothers

**Affiliations:** 1 College of Medicine, Medical University of South Carolina, Charleston, SC USA; 2 Surgical Service, Ralph H. Johnson Department of Veterans Affairs Medical Center, Charleston, SC USA; 3 Division of Vascular and Endovascular Surgery, Department of Surgery, Medical University of South Carolina, Charleston, SC USA

**Keywords:** Vascular Surgical Procedure, Vascular graft occlusion, Peripheral vascular diseases, Vascular patency

## Abstract

**Purpose:**

Blind tunneling of subfascial femoropopliteal bypass grafts may result in inadvertent graft passage through the sartorius. The purpose of this study was to determine whether intramuscular passage of femoropopliteal bypass grafts affects primary patency.

**Methods:**

Patients undergoing femoropopliteal bypass at a Veterans Administration hospital and associated university medical center over a recent 13-year period who also had postoperative cross-sectional imaging adequate to determine graft location were examined. Five-year primary patency of grafts circumferentially enveloped by the muscle was compared with that of both extramuscular subfascial grafts and subcutaneous grafts.

**Results:**

370 femoropopliteal grafts were identified, among which 258 (70%) were subfascial. Vein grafts comprised 51% of the subfascial grafts, and 53% were inserted above the knee. Available postoperative imaging in 110 subfascial grafts demonstrated 74 (67%) to lie completely within the muscle at some point. Among imaged subfascial grafts, primary patency at five years for intramuscular grafts was not significantly worse than extramuscular grafts (*P* = 0.31). This remained true whether grafts were vein (*P* = 0.39) or prosthetic (*P* = 0.31) and whether grafts inserted to the above-knee (*P* = 0.43) or below-knee (*P* = 0.21) popliteal artery. Multivariable Cox regression revealed a significant relationship between use of vein grafts (*P* = 0.013), active smoking (*P* = 0.01), and hypertension (*P* = 0.041) and primary patency, but not intramuscular graft location (*P* = 0.31).

**Conclusion:**

This study failed to demonstrate significantly inferior primary patency among subfascial femoropopliteal grafts tunneled intramuscularly. Larger studies may be required to adequately detect any differences in patency by muscular entrapment, especially among subgroups.

**Supplementary Information:**

The online version contains supplementary material available at 10.1007/s00423-024-03470-1.

## Introduction

Femoropopliteal bypass surgery is currently one of the most common procedures done to relieve claudication in the setting of peripheral vascular disease (PVD), especially in the setting of chronic limb threatening ischemia (CLTI) [1]. During this procedure, the graft may be tunneled subcutaneously or more anatomically in a subfascial plane deep to the sartorius muscle and adjacent to the native arteries [2, 3]. This tunneling, typically done blindly, could result in potential entrapment of the graft within the sartorius muscle [4]. Previous studies have shown that popliteal artery passage through or adjacent to the local musculature can lead to compression and thrombosis of an artery from contraction of the muscle such as occurs with popliteal artery entrapment syndrome or thoracic outlet syndromes [4–6]. However, there is currently insufficient data to demonstrate whether graft passage within the sartorius muscle results in similar outcomes.

Determination of whether a subfascial graft passes through the sartorius or remains external to it cannot be determined by standard arteriography but is discoverable by three-dimensional imaging such as computed tomography (CT). The objective of the current study was to correlate graft patency for femoropopliteal bypass with potential graft passage through the sartorius muscle by retrospective cohort analysis of patients with prior bypass who subsequently underwent CT imaging for any reason. The hypothesis to be tested was that intramuscular passage of a femoropopliteal bypass would be associated with inferior primary patency at five years due to external compression of the graft by the muscle.

## Methods

### Patient population

This retrospective cohort observational comparison of the results of femoropopliteal bypass grafts among patients treated at a Veterans Administration Hospital and University Medical Center was approved by the Research and Development Committee and Institutional Review Board of the respective institutions with waiver of informed consent. Patients who had undergone femoropopliteal bypass for ischemic peripheral vascular disease, generally as defined by a Rutherford stage IIb or greater, during the period of January 2010 to March 2023 were identified.

Medical records were reviewed for demographic and health-related factors, indication for intervention, and graft patency including need for reoperation of the affected area. For post-operative surveillance, patients were seen at two weeks for wound control, followed by three months and then annually thereafter unless concerns or symptoms were raised by the patient. At each postoperative visit later than two weeks, each patient received ankle-brachial index (ABI) measurements and arterial duplex ultrasound. Any potential graft stenosis shown by these two tests was confirmed by computed tomography and/or standard digital subtraction arteriography. Primary patency was defined as the lack of any corrective action performed due to evidence of stenosis and freedom from graft occlusion. Reintervention was performed for greater than 75% stenosis or occlusion as clinically appropriate based on severity of symptoms and clinical condition.

### Surgical protocol

During the femoropopliteal bypass procedure, tunnelling was performed blindly, with the Crawford-Cooley graft tunneling device passed from distal to proximal. When initiated from the above-knee popliteal incision, the tunneler was inserted just deep to the sartorius and blindly pushed proximally, with the same surgeon’s opposite hand placed in the groin incision at the target location to assist in palpating the end of the tunneler as it reached its desired destination alongside the proximal superficial femoral artery. When tunneling of the graft was initiated from the below-knee incision, a tunnel was first manually created digitally between the heads of the gastrocnemius muscle, then passed blindly to the surgeon’s opposed hand as noted above. Neither fluoroscopy nor ultrasound were employed to assist with placement. The choice of tunneling in the subfascial or subcutaneous plane had not been standardized according to graft type or whether primary or secondary procedure during this study. Patients who receives externally reinforced grafts were excluded from this study.

Prior to surgery, all patients were prescribed antiplatelet therapy consisting of aspirin 81 mg daily as well as a high-dose statin per current standard of care [7, 8]. Intraoperatively, patients were given systemic unfractionated heparin during vascular clamping. Post-operatively, patients received clopidogrel 75 mg daily for three months in addition to the long-term management with aspirin 81 mg and high-dose statin. If patients were unable to tolerate aspirin, they were continued on clopidogrel past the usual three-month period. Compliance by the patients with these recommendations was incompletely documented in the record. No additional anticoagulation was administered during the postoperative period.

### Imaging analysis

The existence of any postoperative CT imaging with contrast that would image the affected limb was sought and examined for all patients. In those patients with suitable postoperative CT imaging in which graft location could be assessed, graft location as it traversed the anterior thigh was noted and was defined as intramuscular only in the presence of complete circumferential integration within the sartorius or surrounding musculature at some point along its course. All imaging was reviewed independently by the two authors, and any discrepancies in the assessment of the overall graft location were resolved by combined review. Because intravascular contrast was given during CT imaging for all patients, such imaging was not used to confirm patency.

### Statistical analysis

Regarding the outcome of primary patency, demographic and risk factors were compared with a univariate logistic regression. Variables with a log-rank test *P* < 0.10 on univariate analysis and additional variables judged to be of clinical significance were entered into a Cox multivariate regression model, reported as hazard ratios (HRs) and 95% confidence intervals. Categorical variable bivariate Pearson Correlation Coefficients were checked for multicollinearity using a Pearson Correlation Coefficient value of 0.6 as a threshold. A two-tailed *P*-value of < 0.05 was considered statistically significant. Graft primary patency was analyzed with Kaplan-Meier life table analysis using the log-rank test. Statistical software consistent of SPSS Statistics Version 28.0.1.0 (Armonk, NY: IBM Corporation, 2021).

## Results

**Fig. 1:**
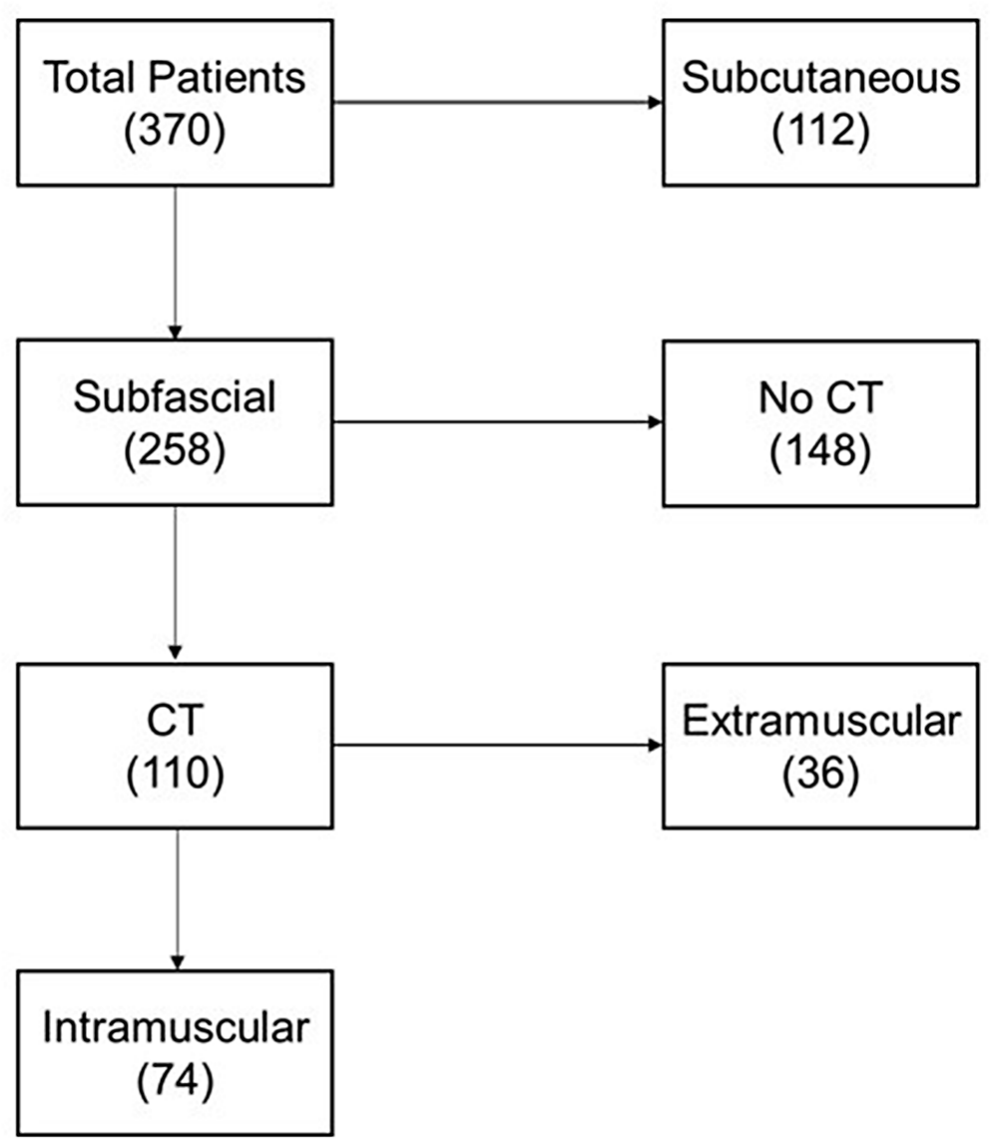
Distribution of study patients

A total of 370 patients who received femoropopliteal grafts were identified, with a demographic distribution as described in Table  1. Among these grafts, 258 (69.7%) were subfascial as noted in operative reports. Vein grafts comprised 51.3% of the total, and 53.2% were inserted to the popliteal artery above the knee. Available postoperative imaging in 110 subfascial grafts demonstrated 74 (67%) to lie completely within the muscle at some point, as demonstrated in Fig. 1. Graft entrapment, when it occurred, was observed primarily in the sartorius muscle, with fewer than 5% in the adductor longus or adductor magnus.

**Table 1:** Description of patient groups

Group	*N*	Imaged	Subfasc*	IM†	Male	Black	Age	Tob ‡	HTN §	NIDDM[]	IDDM ||	COPD¶	CRI#	Elect**	CLTI ††	AK^a^	Prosth^b^
All patients	370	38%	70%	-	86%	32%	64.4 *±* 8.8	25%	80%	20%	18%	11%	5%	76%	47%	53%	49%
Not imaged	231	0%	65%	-	87%	26%	67.0 *±* 8.8	29%	80%	21%	21%	12%	6%	75%	45%	52%	45%
Imaged	139	100%	78%	53%	84%	41%	65.4 *±* 8.9	22%	81%	19%	14%	10%	4%	77%	49%	55%	55%
Subcut ^c^	31	100%	0%	-	81%	45%	66.9 *±* 8.0	23%	81%	23%	10%	13%	3%	77%	50%	19%	52%
Subfasc*	108	100%	100%	68%	85%	40%	65.0 *±* 9.1	21%	81%	18%	15%	9%	4%	77%	49%	66%	56%
EM^d^	34	100%	100%	0%	82%	41%	67.3 *±* 8.3	26%	79%	15%	18%	3%	3%	76%	53%	47%	53%
IM†	74	100%	100%	100%	86%	39%	63.9 *±* 9.3	19%	81%	19%	14%	12%	4%	77%	46%	74%	58%

*Subfascial.

†Intramuscular.

‡Current smoking.

§Hypertension.

[]Non-insulin dependent diabetes mellitus.

||Insulin-dependent diabetes mellitus.

¶Chronic obstructive pulmonary disease.

#Chronic renal insufficiency.

**Elective procedure.

††Chronic limb-threatening ischemia with rest pain or tissue loss *(Rutherford 4–6 or Fontaine III to IV)*.

^a^Above knee popliteal distal anastomosis.

^b^Prosthetic graft.

^c^Subcutaneous.

Five-year primary patency was significantly worse for grafts in which follow-up imaging had been performed (37% vs. 54%, *P* = 0.0087), as questions of patency or planning for revision/revascularization likely prompted such imaging. Among patent subfascial grafts, the proportion that were intramuscular (68%, or 65% among those at least 5 years old) did not differ from the proportion of non-patent grafts (67%). This finding is consistent with the impression that acquisition of postoperative imaging was not biased by preferentially imaging intramuscular grafts. There was no significant difference in five-year primary patency for grafts tunneled subcutaneously compared to those tunneled in the subfascial plane (42% vs. 44%, *p* = 0.67). Small differences in patency between bypasses inserted into the above-knee vs. below-knee popliteal artery (46% vs. 38%, *p* = 0.12) did not attain statistical significance. None of below-knee grafts with available imaging showed muscular passage in the P1, P2, or P3 segments of the popliteal artery, suggesting that entrapment by the gastrocnemius did not occur.

Among imaged subfascial grafts, intramuscular graft location did not lead to inferior primary patency at five years compared with other non-intramuscular grafts (32% vs. 39%, *P* = 0.31). This remained true whether grafts consisted of vein (54% vs. 53%, *P* = 0.39) or prosthetic (12% v. 25%, *P* = 0.31) and whether grafts extended to the above-knee (38% vs. 42%, *P* = 0.43) or below-knee (10% vs. 35%, *P* = 0.21) popliteal artery. The already small sample size and likelihood of Type 2 statistical errors limited further sub-analysis as to whether prosthetic below-knee popliteal grafts were compromised by intramuscular tunneling (16 of 27 intramuscular, 0% vs. 24%, *P* = 0.14). Similarly, there were no differences in assisted primary patency, secondary patency, or limb salvage between intramuscular and extramuscular grafts at five years within each of the subgroups above (Table 2).

**Table 2:** Assisted primary patency, secondary patency, and limb salvage of imaged subfascial grafts according to intramuscular location.

	Assisted Primary Patency				Secondary Patency				Limb Salvage		
	IM*	EM†	P	IM*		EM†	P	IM*		EM†	P
All	40%	41%	0.37	53%		47%	0.23	88%		80%	0.17
Prosthetic	13%	25%	0.46	36%		40%	0.35	82%		71%	0.25
Vein	69%	57%	0.22	69%		59%	0.22	93%		88%	0.32
Above knee	46%	45%	0.50	54%		47%	0.41	98%		94%	0.32
Below knee	13%	35%	0.36	40%		47%	0.37	80%		68%	0.24
Below knee prosthetic	0%	24%	0.21	33%		44%	0.43	77%		61%	0.29

*Intramuscular.

†Extramuscular

Univariate analysis of various risk factors for loss of primary patency revealed that the use of vein graft (HR = 0.47, *P* = 0.003) and active smoking status (HR = 2.02, *P* = 0.009) significantly affected the primary patency of the femoropopliteal grafts. (Table 3). However, there was no significant difference between those grafts tunneled intramuscularly compared with those tunneled outside the muscle (HR = 1.02, *P* = 0.94). This was confirmed with multivariable Cox regression, which revealed use of vein graft (HR = 0.51, *P* = 0.013), smoking status (HR = 2.8, *P* = 0.01), and hypertension (HR = 2.0, *P* = 0.041) but not intramuscular graft location (HR = 1.3, *P* = 0.31) to be predictive of primary patency (Table 4).

**Table 3:** Univariate analysis

Variable	Hazard Ratio	95% CI	*P*
**Vein Graft**	**0.47**	**0.028-0.77**	**0.003**
**Active Smoking**	**2.02**	**1.19–3.42**	**0.009**
Hypertension	1.75	0.92–3.33	0.088
Urgent/Emergent	1.55	0.93–2.58	0.090
Below knee target	1.44	0.90–2.30	0.13
COPD	1.64	0.74–3.63	0.22
Age	1.12	0.99–1.04	0.24
Caucasian race	0.84	0.53–1.34	0.46
Male sex	0.87	0.46–1.66	0.67
Renal insufficiency	1.21	0.30–4.94	0.80
Diabetes mellitus	1.03	0.63–1.66	0.92
*Intramuscular*	*1.02*	*0.63–1.65*	*0.94*

*Confidence interval.

†Chronic obstructive pulmonary disease.

**Table 4:** Multivariate analysis

Variable	Hazard Ratio	95% CI*	*P*
Active Smoking	2.83	1.55–5.19	< 0.001
Vein Graft	0.51	0.30–0.87	0.013
Hypertension	2.00	1.03–3.89	0.041
Below knee target	1.51	0.89–2.59	0.13
Intramuscular	1.33	0.77–2.28	0.31
Urgent/Emergent	1.00	0.57–1.75	0.99

*Confidence interval.

**Fig. 2:**
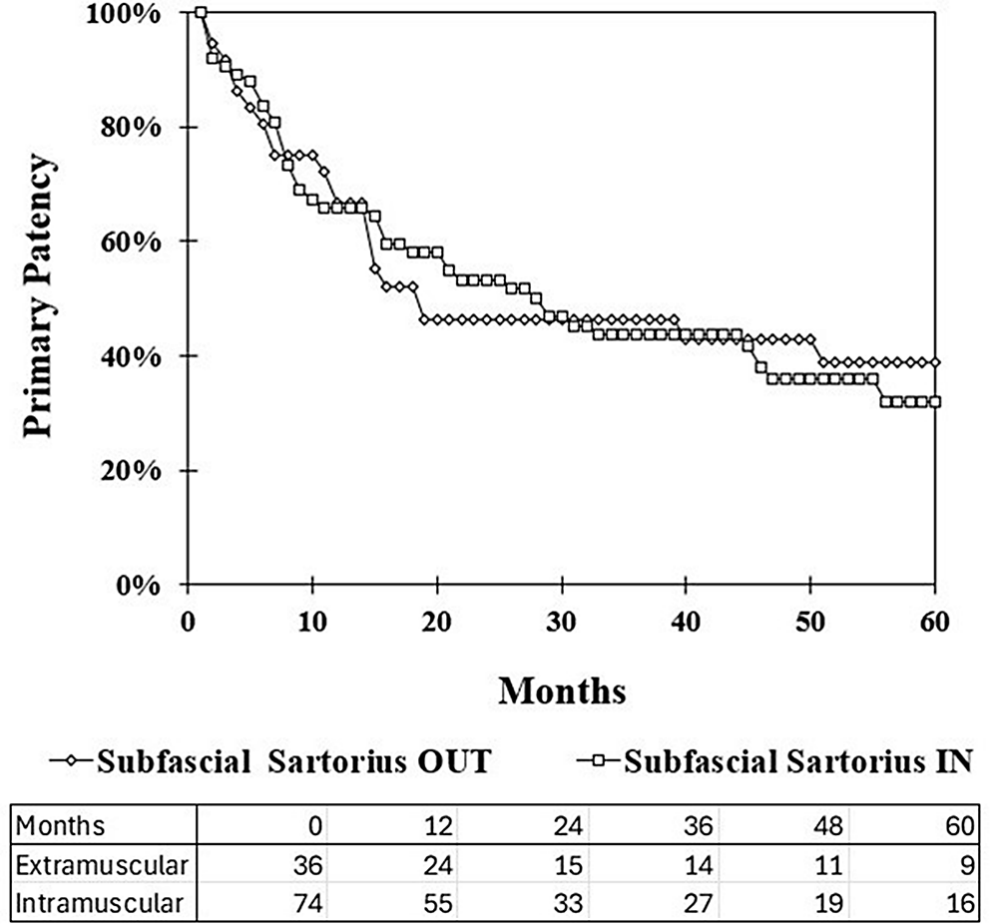
Kaplan-Meyer analysis for primary patency of extramuscular and intramuscular subfascial femoropopliteal bypass graft to 60 months. The corresponding life table is shown below, with *p* = 0.48 and z = 0.061. Standard error remains < 0.10 through 60 months

These findings were further confirmed when analyzing the primary patency through Kaplan-Meyer analysis, as shown in Fig. 2, which demonstrates no significant difference in 5-year primary patency between intramuscular and extramuscular subfacial grafts (*p* = 0.48). When analyzing the outcomes of extramuscular versus intramuscular grafts, any potential initially greater decrease in overall primary patency for grafts tunneled outside of the muscle within the first 20 months did not persist over the longer interval.

## Discussion

The concept of muscular compression of vascular structures leading to injury or occlusion is not unique. In addition to the well-described syndrome of popliteal entrapment, compression of the subclavian artery by abnormal or accessory slips of the anterior scalene may lead to arterial stenosis, post-stenotic dilation, and eventual occlusion [5, 9]. Before the advent of high-resolution three-dimensional vascular imaging, simple arteriography in two dimensions would likely be expected to fail to detect intramuscular location of femoropopliteal bypass in the relaxed muscle except in situations of extreme external constriction. Although potentially detectable with careful duplex imaging, intramuscular passage of a subfascial graft was easily determined on cross-sectional CT imaging. Whether such unintended intramuscular graft location might affect the patency outcomes had not been subject to investigation. The current study represents an attempt to at least introduce the discussion. The current analysis focuses on primary patency, and while arguably not the most important outcome for patient impact and quality of life, this outcome was felt to be the most appropriate for determining the impact of external graft compression on the conduit [10]. In fact, multivariable Cox regression analysis of the effect of graft muscular location failed to show any effect on assisted primary patency, secondary patency, limb salvage, major adverse limb event, or amputation-free survival.

Numerous other factors are known to affect long term patency of femoropopliteal bypass grafts. Autogenous great saphenous vein has been repeatedly demonstrated superior to prosthetic, homograft, short saphenous vein, and cephalic/basilic vein. Grafts anastomosed to the popliteal artery above the knee enjoy better patency than those extending below the knee [11, 12]. By contrast, use of an adequate great saphenous vein in the reversed configuration was not demonstrated to have different patency from great saphenous in the in-situ location [12]. Such observations are supported by the findings of the current study. Another potential cause of graft failure might be infection [13]. Unfortunately, in our study the incidence of prosthetic graft infection as a cause of graft failure was not recorded at the time of chart review, and removal of linkage data per institutional protocol during the study now prevents further retroactive analysis. This also applies to the incidence of perioperative wound issues such as hematoma or anastomotic pseudoaneurysm formation, which would also present a potential need for reoperation or graft revision.

In general, this study has considered graft anatomic position as it relates to unintentional muscular compression as a potential risk factor for future graft occlusion. However, complete evaluation of potential effect of subfascial graft location in certain subsets of the population in this study was limited by the small number of grafts placed in the various combinations of prosthetic and vein grafts and above/below knee popliteal targets. The sample size remains far too small to confirm any potential adverse effect of intramuscular passage for prosthetic grafts extending below the knee, with any statistical confidence.

Other limitations of the present study should be considered. Although the determination of graft location was completed by co-review of the related imaging by only two authors, potential areas for error were mitigated by multiple cross-review by the authors during each of the data transfer steps. Another limitation of this study is the loss of follow-up for some patients, which may be more common for patients who are asymptomatic and do not feel the need to continue to follow up, while patients who experience recurrence of symptoms may prove to be most likely to follow up with their vascular surgeon. An additional limitation is that because of the lack of standardization for the tunneling process for femoropopliteal bypass grafts and intraoperative imaging, this study relies on institutional protocol and experience. Finally, due to the potential limitations of our institutional medical records system, it was not possible to determine whether patients subsequently underwent operations at outside institutions.

## Conclusion

This study fails to demonstrate significant differences in primary patency according to whether subfascial femoropopliteal grafts pass through the sartorius muscle. Interestingly, most subfascial grafts were tunneled intramuscularly at some point. Larger and, ideally, multi-institutional studies may be required to adequately detect any differences in patency by muscular entrapment, especially for prosthetic grafts to the below knee popliteal artery. Such further analysis might be useful to address current guidelines regarding femoropopliteal bypass graft tunneling strategies for peripheral artery occlusion, including any potential beneficial utility of intraoperative imaging with the purpose of detecting graft tunneling location.

## Electronic supplementary material

Below is the link to the electronic supplementary material.

Supplementary Material 1

## Data Availability

Data is provided within the supplementary information file.
